# 6-(4-Bromo­phen­yl)-2-eth­oxy-4-(4-ethoxy­phen­yl)nicotinonitrile

**DOI:** 10.1107/S1600536809051861

**Published:** 2009-12-09

**Authors:** Suchada Chantrapromma, Hoong-Kun Fun, Thitipone Suwunwong, Mahesh Padaki, Arun M. Isloor

**Affiliations:** a Crystal Materials Research Unit, Department of Chemistry, Faculty of Science, Prince of Songkla University, Hat-Yai, Songkhla 90112, Thailand; b X-ray Crystallography Unit, School of Physics, Universiti Sains Malaysia, 11800 USM, Penang, Malaysia; c Department of Chemistry, National Institute of Technology-Karnataka, Surathkal, Mangalore 575 025, India

## Abstract

The mol­ecule of the title nicotinonitrile derivative, C_22_H_19_BrN_2_O_2_, is non-planar, the central pyridine ring making dihedral angles of 7.34 (14) and 43.56 (15)° with the 4-bromo­phenyl and 4-ethoxy­phenyl rings, respectively. The eth­oxy group of the 4-ethoxy­phenyl is slightly twisted from the attached benzene ring [C—O—C—C = 174.2 (3)°], whereas the eth­oxy group attached to the pyridine ring is in a (+)*syn*-clinal conformation [C—O—C—C = 83.0 (3)°]. A weak intra­molecular C—H⋯N inter­action generates an *S*(5) ring motif. In the crystal structure, the mol­ecules are linked by weak inter­molecular C—H⋯N inter­actions into screw chains along the *b* axis. These chains stacked along the *a* axis. π–π inter­actions with centroid–centroid distances of 3.8724 (16) and 3.8727 (16) Å are also observed.

## Related literature

For bond-length data, see: Allen *et al.* (1987 ▶). For hydrogen-bond motifs, see: Bernstein *et al.* (1995 ▶). For the synthesis and applications of nicotinonitrile derivatives, see: Borgna *et al.* (1993 ▶); Fun *et al.* (2008 ▶); Goda *et al.* (2004 ▶); Kamal *et al.* (2007 ▶); Malinka *et al.* (1998 ▶). For related structures, see: Chantrapromma *et al.* (2009 ▶). For the stability of the temperature controller used in the data collection, see Cosier & Glazer (1986 ▶).
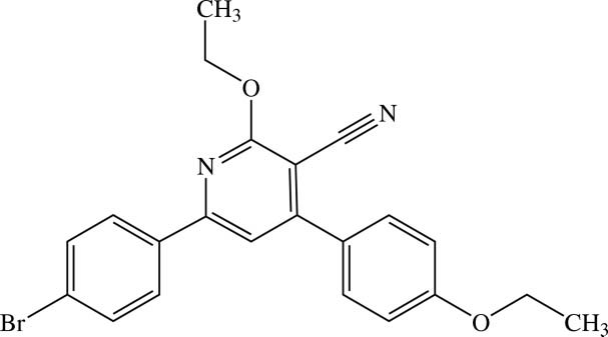


## Experimental

### 

#### Crystal data

C_22_H_19_BrN_2_O_2_
*M* *_r_* = 423.29
Orthorhombic, 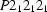

*a* = 4.3414 (2) Å
*b* = 14.7392 (6) Å
*c* = 29.4409 (13) Å
*V* = 1883.89 (14) Å^3^
*Z* = 4
Mo *K*α radiation
μ = 2.20 mm^−1^
*T* = 100 K
0.57 × 0.05 × 0.03 mm

#### Data collection

Bruker APEXII CCD area-detector diffractometer
Absorption correction: multi-scan (*SADABS*; Bruker, 2005 ▶) *T*_min_ = 0.368, *T*_max_ = 0.931
18389 measured reflections
5477 independent reflections
4081 reflections with *I* > 2σ(*I*)
*R*_int_ = 0.076

#### Refinement

*R*[*F* ^2^ > 2σ(*F* ^2^)] = 0.039
*wR*(*F* ^2^) = 0.084
*S* = 0.99
5477 reflections
246 parameters
H-atom parameters constrained
Δρ_max_ = 0.58 e Å^−3^
Δρ_min_ = −0.54 e Å^−3^
Absolute structure: Flack (1983 ▶), 2269 Friedel pairs
Flack parameter: 0.008 (9)

### 

Data collection: *APEX2* (Bruker, 2005 ▶); cell refinement: *SAINT* (Bruker, 2005 ▶); data reduction: *SAINT*; program(s) used to solve structure: *SHELXTL* (Sheldrick, 2008 ▶); program(s) used to refine structure: *SHELXTL*; molecular graphics: *SHELXTL*; software used to prepare material for publication: *SHELXTL* and *PLATON* (Spek, 2009 ▶).

## Supplementary Material

Crystal structure: contains datablocks global, I. DOI: 10.1107/S1600536809051861/sj2683sup1.cif

Structure factors: contains datablocks I. DOI: 10.1107/S1600536809051861/sj2683Isup2.hkl

Additional supplementary materials:  crystallographic information; 3D view; checkCIF report

## Figures and Tables

**Table 1:** Hydrogen-bond geometry (Å, °)

*D*—H⋯*A*	*D*—H	H⋯*A*	*D*⋯*A*	*D*—H⋯*A*
C1—H1*A*⋯N1	0.93	2.41	2.758 (4)	102
C5—H5*A*⋯N2^i^	0.93	2.58	3.446 (4)	156
C13—H13*A*⋯N2^ii^	0.93	2.53	3.206 (4)	130

Symmetry codes: (i) 
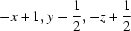
; (ii) 
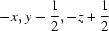
.

**Fig. 1.**
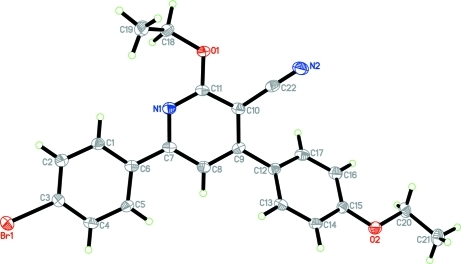
The molecular structure of the title compound, showing 50% probability displacement ellipsoids and the atom-numbering scheme.

**Fig. 2.**
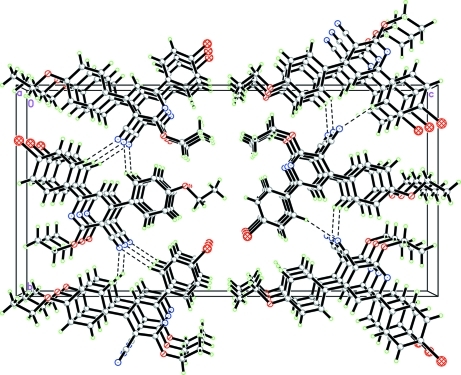
The crystal packing of the title compound viewed along the a axis, showing chains stacked down the a axis. Hydrogen bonds are shown as dashed lines.

## supplementary crystallographic information

### Comment

A large number of substituted pyridines have been claimed to exhibit biological activities in a number of areas (Borgna *et al.*, 1993; Goda *et al.*, 2004; Kamal *et al.*, 2007; Malinka *et al.*, 1998). The pyridine ring is among the most common heterocyclic compounds found in the naturally occurring heterocycles and in various therapeutic agents. Our research is aimed at the synthesis and preliminary pharmacological screening (*in vivo*) of the nicotinonitrile derivatives. Therefore the title nicotinonitrile derivative, which is a substituted pyridine compound, was synthesized by cyclization of a chalcone derivative (Fun *et al.*, 2008) and malononitrile in order to investigate its analgesic and anti-inflammatory activities. Our results of these pharmacological studies showed that the title compound is a promising candidate for analgesic and anti-inflammatory activities. The analgesic and anti-inflammatory profiles of the title compound together with some other related nicotinonitrile derivatives will be reported elsewhere.

The title compound (I), C_22_H_19_BrN_2_O_2_ is a non-planar molecule (Fig. 1). The central pyridine ring is nearly coplanar with the 4-bromophenyl ring with the dihedral angle of 7.34 (14)° whereas it is inclined to the 4-ethoxyphenyl unit with the dihedral angle of 43.56 (15)°. The ethoxy substituent of the 4-ethoxyphenyl is slightly twisted from the mean plane of the attached benzene ring with the torsion angle C15–O2–C20–C21 = 174.2 (3)° whereas the ethoxy group attached to the pyridine ring is in a (+)*syn*-clinal conformation with a C11–O1–C18–C19 torsion angle of 83.0 (3)°. The orientation of the cyano group can be indicated by the torsion angle C8–C9–C10–C22 = 177.0 (3)°. A weak intramolecular C1—H1A···N1 interaction generates an S(5) ring motif (Bernstein *et al.*, 1995). The bond distances agree with the literature values (Allen *et al.*, 1987) and are comparable with those for a related structure (Chantrapromma *et al.*, 2009).

In the crystal structure (Fig. 2), the molecules are linked by weak intermolecular C—H···N interactions (Table 1) into screw chains along the *b* axis. These chains stacked along the *a* axis. The crystal is further stabilized by π···π interactions with the *Cg*_1_···*Cg*_2_ distances of 3.8724 (16) Å (symmetry code: -1 + *x*, *y*, *z*) and 3.8727 (16) Å (symmetry code: 1 + *x*, *y*, *z*); *Cg*_1_ and *Cg*_2_ are the centroids of C7–C11/N1 and C1–C6 rings, respectively.

### Experimental

(*E*-1-(4-Bromophenyl)-3-(4-ethoxyphenyl)prop-2-en-1-one (0.50 g, 0.0015 mole) were added with continuous stirring to a freshly prepared sodium alkoxide (0.0014 mole of sodium in 100 ml of ethanol). Malononitrile (1.30 g, 0.02 mol) was then added with continuous stirring at room temperature until the precipitate separated out. The resulting solid was filtered (yield 65%). Colorless needle-shaped single crystals of the title compound suitable for *x*-ray structure determination were recrystalized from acetone/ethanol (1:1 *v*/*v*) by the slow evaporation of the solvent at room temperature over several days, Mp. 418–419 K.

### Refinement

All H atoms were positioned geometrically and allowed to ride on their parent atoms, with d(C—H) = 0.93 Å for aromatic, 0.97 for CH_2_ and 0.96 Å for CH_3_ atoms. The *U*_iso_ values were constrained to be 1.5*U*_eq_ of the carrier atom for methyl H atoms and 1.2*U*_eq_ for the remaining H atoms. A rotating group model was used for the methyl groups. The highest residual electron density peak is located at 1.07 Å from Br1 and the deepest hole is located at 0.96 Å from Br1.

### Crystal data

**Table:**

C_22_H_19_BrN_2_O_2_	*D*_x_ = 1.492 Mg m^−^^3^
*M**_r_* = 423.29	Melting point = 418–419 K
Orthorhombic, *P*2_1_2_1_2_1_	Mo *K*α radiation, λ = 0.71073 Å
Hall symbol: P 2ac 2ab	Cell parameters from 5478 reflections
*a* = 4.3414 (2) Å	θ = 1.4–30.0°
*b* = 14.7392 (6) Å	µ = 2.20 mm^−^^1^
*c* = 29.4409 (13) Å	*T* = 100 K
*V* = 1883.89 (14) Å^3^	Needle, colourless
*Z* = 4	0.57 × 0.05 × 0.03 mm
*F*(000) = 864	

### Data collection

**Table:**

Bruker APEXII CCD area detector diffractometer	5477 independent reflections
Radiation source: sealed tube	4081 reflections with *I* > 2σ(*I*)
graphite	*R*_int_ = 0.076
φ and ω scans	θ_max_ = 30.0°, θ_min_ = 1.6°
Absorption correction: multi-scan (*SADABS*; Bruker, 2005)	*h* = −6→6
*T*_min_ = 0.368, *T*_max_ = 0.931	*k* = −20→20
18389 measured reflections	*l* = −41→41

### Refinement

**Table:**

Refinement on *F*^2^	Secondary atom site location: difference Fourier map
Least-squares matrix: full	Hydrogen site location: inferred from neighbouring sites
*R*[*F*^2^ > 2σ(*F*^2^)] = 0.039	H-atom parameters constrained
*wR*(*F*^2^) = 0.084	*w* = 1/[σ^2^(*F*_o_^2^) + (0.0276*P*)^2^] where *P* = (*F*_o_^2^ + 2*F*_c_^2^)/3
*S* = 0.99	(Δ/σ)_max_ = 0.001
5477 reflections	Δρ_max_ = 0.58 e Å^−^^3^
246 parameters	Δρ_min_ = −0.54 e Å^−^^3^
0 restraints	Absolute structure: Flack (1983), 2269 Friedel pairs
Primary atom site location: structure-invariant direct methods	Flack parameter: 0.008 (9)

### Special details

**Table:**

Experimental. The crystal was placed in the cold stream of an Oxford Cryosystems Cobra open-flow nitrogen cryostat (Cosier & Glazer, 1986) operating at 100.0 (1) K.
Geometry. All e.s.d.'s (except the e.s.d. in the dihedral angle between two l.s. planes) are estimated using the full covariance matrix. The cell e.s.d.'s are taken into account individually in the estimation of e.s.d.'s in distances, angles and torsion angles; correlations between e.s.d.'s in cell parameters are only used when they are defined by crystal symmetry. An approximate (isotropic) treatment of cell e.s.d.'s is used for estimating e.s.d.'s involving l.s. planes.
Refinement. Refinement of *F*^2^ against ALL reflections. The weighted *R*-factor *wR* and goodness of fit *S* are based on *F*^2^, conventional *R*-factors *R* are based on *F*, with *F* set to zero for negative *F*^2^. The threshold expression of *F*^2^ > σ(*F*^2^) is used only for calculating *R*-factors(gt) *etc*. and is not relevant to the choice of reflections for refinement. *R*-factors based on *F*^2^ are statistically about twice as large as those based on *F*, and *R*- factors based on ALL data will be even larger.

### Fractional atomic coordinates and isotropic or equivalent isotropic displacement parameters (Å^2^)

**Table:**

	*x*	*y*	*z*	*U*_iso_*/*U*_eq_	
Br1	1.58148 (8)	0.282863 (19)	0.048491 (10)	0.02362 (8)	
O1	0.4205 (6)	0.74788 (11)	0.14589 (6)	0.0216 (4)	
O2	−0.0548 (6)	0.47728 (13)	0.40035 (6)	0.0252 (5)	
N2	0.0125 (7)	0.76955 (17)	0.24500 (8)	0.0298 (7)	
N1	0.7019 (6)	0.61534 (15)	0.14495 (8)	0.0196 (5)	
C1	1.0999 (8)	0.50365 (18)	0.09800 (9)	0.0197 (6)	
H1A	1.0551	0.5618	0.0877	0.024*	
C2	1.2818 (8)	0.44749 (19)	0.07176 (10)	0.0214 (7)	
H2A	1.3606	0.4679	0.0442	0.026*	
C3	1.3459 (7)	0.36055 (18)	0.08677 (9)	0.0191 (7)	
C4	1.2361 (8)	0.32993 (19)	0.12783 (10)	0.0220 (7)	
H4A	1.2821	0.2716	0.1377	0.026*	
C5	1.0564 (8)	0.38691 (17)	0.15427 (9)	0.0204 (6)	
H5A	0.9838	0.3665	0.1822	0.025*	
C6	0.9818 (7)	0.47489 (18)	0.13976 (9)	0.0185 (7)	
C7	0.7817 (7)	0.53544 (18)	0.16580 (10)	0.0178 (6)	
C8	0.6704 (7)	0.51430 (19)	0.20904 (10)	0.0195 (7)	
H8A	0.7262	0.4597	0.2226	0.023*	
C9	0.4766 (7)	0.57403 (18)	0.23214 (9)	0.0170 (6)	
C10	0.3912 (8)	0.65388 (18)	0.20974 (9)	0.0189 (6)	
C11	0.5132 (7)	0.67004 (18)	0.16607 (9)	0.0186 (7)	
C12	0.3562 (8)	0.55145 (19)	0.27789 (9)	0.0189 (7)	
C13	0.2478 (8)	0.46338 (18)	0.28616 (10)	0.0197 (7)	
H13A	0.2680	0.4192	0.2638	0.024*	
C14	0.1114 (9)	0.44126 (18)	0.32705 (9)	0.0200 (7)	
H14A	0.0369	0.3829	0.3318	0.024*	
C15	0.0855 (9)	0.50606 (18)	0.36103 (9)	0.0199 (6)	
C16	0.1977 (8)	0.5937 (2)	0.35411 (10)	0.0230 (7)	
H16A	0.1834	0.6371	0.3769	0.028*	
C17	0.3314 (7)	0.61494 (19)	0.31246 (10)	0.0222 (7)	
H17A	0.4061	0.6733	0.3077	0.027*	
C18	0.5818 (8)	0.7761 (2)	0.10480 (8)	0.0230 (6)	
H18A	0.7994	0.7626	0.1081	0.028*	
H18B	0.5605	0.8412	0.1012	0.028*	
C19	0.4616 (8)	0.7296 (2)	0.06258 (9)	0.0284 (7)	
H19A	0.5543	0.7564	0.0362	0.043*	
H19B	0.2420	0.7366	0.0610	0.043*	
H19C	0.5123	0.6662	0.0637	0.043*	
C20	−0.1371 (8)	0.5444 (2)	0.43323 (10)	0.0243 (8)	
H20A	−0.2638	0.5911	0.4195	0.029*	
H20B	0.0460	0.5726	0.4458	0.029*	
C21	−0.3148 (8)	0.4952 (2)	0.46998 (11)	0.0306 (8)	
H21A	−0.3648	0.5368	0.4939	0.046*	
H21B	−0.1911	0.4467	0.4819	0.046*	
H21C	−0.5011	0.4707	0.4574	0.046*	
C22	0.1818 (7)	0.7186 (2)	0.22906 (9)	0.0210 (6)	

### Atomic displacement parameters (Å^2^)

**Table:**

	*U*^11^	*U*^22^	*U*^33^	*U*^12^	*U*^13^	*U*^23^
Br1	0.02481 (15)	0.02126 (12)	0.02480 (13)	0.00135 (15)	−0.00071 (16)	−0.00381 (13)
O1	0.0280 (11)	0.0181 (9)	0.0186 (9)	0.0039 (10)	0.0014 (12)	0.0043 (7)
O2	0.0376 (14)	0.0207 (9)	0.0173 (9)	0.0022 (11)	0.0044 (11)	−0.0004 (8)
N2	0.040 (2)	0.0236 (13)	0.0260 (12)	0.0087 (13)	−0.0018 (12)	0.0006 (10)
N1	0.0204 (14)	0.0189 (11)	0.0195 (12)	−0.0008 (11)	−0.0024 (11)	0.0008 (9)
C1	0.0211 (16)	0.0187 (12)	0.0192 (13)	0.0005 (15)	−0.0017 (15)	0.0004 (10)
C2	0.0186 (16)	0.0227 (15)	0.0229 (15)	−0.0008 (14)	−0.0008 (14)	0.0022 (12)
C3	0.0169 (18)	0.0196 (13)	0.0209 (14)	−0.0009 (12)	−0.0019 (13)	−0.0023 (11)
C4	0.0259 (18)	0.0154 (13)	0.0246 (16)	0.0012 (13)	−0.0041 (15)	0.0017 (11)
C5	0.0233 (17)	0.0196 (13)	0.0184 (13)	0.0003 (15)	−0.0004 (15)	0.0035 (10)
C6	0.0169 (18)	0.0186 (13)	0.0199 (14)	−0.0010 (12)	−0.0061 (13)	0.0020 (11)
C7	0.0172 (16)	0.0152 (13)	0.0210 (15)	−0.0022 (12)	−0.0055 (13)	0.0014 (11)
C8	0.0186 (19)	0.0190 (14)	0.0208 (15)	0.0006 (13)	−0.0027 (13)	0.0010 (12)
C9	0.0161 (17)	0.0181 (12)	0.0167 (13)	−0.0037 (12)	−0.0049 (12)	0.0021 (10)
C10	0.0219 (16)	0.0165 (12)	0.0182 (13)	0.0004 (14)	−0.0045 (14)	−0.0016 (10)
C11	0.0203 (19)	0.0141 (12)	0.0213 (13)	−0.0008 (12)	−0.0040 (12)	0.0016 (10)
C12	0.023 (2)	0.0186 (13)	0.0157 (14)	−0.0007 (13)	−0.0018 (13)	0.0008 (10)
C13	0.0243 (17)	0.0159 (13)	0.0191 (15)	0.0034 (13)	−0.0033 (14)	−0.0017 (11)
C14	0.0243 (18)	0.0157 (13)	0.0199 (14)	0.0017 (14)	−0.0019 (15)	0.0015 (10)
C15	0.0240 (16)	0.0215 (13)	0.0140 (13)	0.0030 (16)	0.0007 (15)	0.0007 (10)
C16	0.0288 (19)	0.0214 (14)	0.0188 (15)	0.0010 (14)	−0.0027 (15)	−0.0025 (12)
C17	0.0260 (19)	0.0169 (13)	0.0237 (15)	−0.0011 (13)	−0.0053 (14)	0.0001 (11)
C18	0.0266 (15)	0.0221 (13)	0.0204 (13)	−0.0010 (18)	0.0012 (15)	0.0036 (11)
C19	0.0302 (19)	0.0321 (16)	0.0230 (13)	0.0023 (17)	0.0012 (14)	0.0030 (12)
C20	0.029 (2)	0.0276 (15)	0.0165 (13)	0.0029 (15)	0.0022 (14)	−0.0077 (11)
C21	0.033 (2)	0.0360 (18)	0.0228 (16)	−0.0011 (17)	0.0033 (15)	−0.0058 (14)
C22	0.0264 (17)	0.0196 (12)	0.0171 (13)	−0.0023 (16)	−0.0042 (12)	0.0026 (13)

### Geometric parameters (Å, °)

**Table:**

Br1—C3	1.905 (3)	C10—C11	1.411 (4)
O1—C11	1.353 (3)	C10—C22	1.435 (4)
O1—C18	1.458 (3)	C12—C17	1.387 (4)
O2—C15	1.375 (3)	C12—C13	1.402 (4)
O2—C20	1.429 (3)	C13—C14	1.380 (4)
N2—C22	1.151 (4)	C13—H13A	0.9300
N1—C11	1.307 (4)	C14—C15	1.388 (4)
N1—C7	1.372 (3)	C14—H14A	0.9300
C1—C2	1.380 (4)	C15—C16	1.395 (4)
C1—C6	1.398 (4)	C16—C17	1.392 (4)
C1—H1A	0.9300	C16—H16A	0.9300
C2—C3	1.384 (4)	C17—H17A	0.9300
C2—H2A	0.9300	C18—C19	1.512 (4)
C3—C4	1.376 (4)	C18—H18A	0.9700
C4—C5	1.386 (4)	C18—H18B	0.9700
C4—H4A	0.9300	C19—H19A	0.9600
C5—C6	1.403 (4)	C19—H19B	0.9600
C5—H5A	0.9300	C19—H19C	0.9600
C6—C7	1.462 (4)	C20—C21	1.514 (4)
C7—C8	1.397 (4)	C20—H20A	0.9700
C8—C9	1.395 (4)	C20—H20B	0.9700
C8—H8A	0.9300	C21—H21A	0.9600
C9—C10	1.399 (4)	C21—H21B	0.9600
C9—C12	1.483 (4)	C21—H21C	0.9600
			
C11—O1—C18	117.6 (2)	C14—C13—H13A	119.5
C15—O2—C20	117.9 (2)	C12—C13—H13A	119.5
C11—N1—C7	118.4 (3)	C13—C14—C15	120.1 (3)
C2—C1—C6	121.4 (3)	C13—C14—H14A	120.0
C2—C1—H1A	119.3	C15—C14—H14A	120.0
C6—C1—H1A	119.3	O2—C15—C14	115.5 (2)
C1—C2—C3	119.4 (3)	O2—C15—C16	124.3 (2)
C1—C2—H2A	120.3	C14—C15—C16	120.2 (3)
C3—C2—H2A	120.3	C17—C16—C15	118.8 (3)
C4—C3—C2	121.0 (3)	C17—C16—H16A	120.6
C4—C3—Br1	120.6 (2)	C15—C16—H16A	120.6
C2—C3—Br1	118.4 (2)	C12—C17—C16	121.8 (3)
C3—C4—C5	119.3 (3)	C12—C17—H17A	119.1
C3—C4—H4A	120.3	C16—C17—H17A	119.1
C5—C4—H4A	120.3	O1—C18—C19	112.8 (3)
C4—C5—C6	121.3 (3)	O1—C18—H18A	109.0
C4—C5—H5A	119.4	C19—C18—H18A	109.0
C6—C5—H5A	119.4	O1—C18—H18B	109.0
C1—C6—C5	117.6 (3)	C19—C18—H18B	109.0
C1—C6—C7	119.6 (2)	H18A—C18—H18B	107.8
C5—C6—C7	122.8 (3)	C18—C19—H19A	109.5
N1—C7—C8	120.8 (3)	C18—C19—H19B	109.5
N1—C7—C6	116.1 (3)	H19A—C19—H19B	109.5
C8—C7—C6	123.2 (2)	C18—C19—H19C	109.5
C9—C8—C7	120.8 (3)	H19A—C19—H19C	109.5
C9—C8—H8A	119.6	H19B—C19—H19C	109.5
C7—C8—H8A	119.6	O2—C20—C21	106.2 (2)
C8—C9—C10	117.5 (3)	O2—C20—H20A	110.5
C8—C9—C12	120.9 (3)	C21—C20—H20A	110.5
C10—C9—C12	121.6 (3)	O2—C20—H20B	110.5
C9—C10—C11	118.2 (3)	C21—C20—H20B	110.5
C9—C10—C22	122.7 (3)	H20A—C20—H20B	108.7
C11—C10—C22	119.1 (2)	C20—C21—H21A	109.5
N1—C11—O1	120.1 (2)	C20—C21—H21B	109.5
N1—C11—C10	124.4 (3)	H21A—C21—H21B	109.5
O1—C11—C10	115.6 (2)	C20—C21—H21C	109.5
C17—C12—C13	118.1 (3)	H21A—C21—H21C	109.5
C17—C12—C9	122.9 (2)	H21B—C21—H21C	109.5
C13—C12—C9	118.9 (2)	N2—C22—C10	179.0 (3)
C14—C13—C12	120.9 (3)		
			
C6—C1—C2—C3	0.8 (5)	C7—N1—C11—C10	1.9 (4)
C1—C2—C3—C4	−1.3 (5)	C18—O1—C11—N1	−11.6 (4)
C1—C2—C3—Br1	177.1 (2)	C18—O1—C11—C10	168.9 (3)
C2—C3—C4—C5	0.6 (5)	C9—C10—C11—N1	−0.1 (5)
Br1—C3—C4—C5	−177.8 (2)	C22—C10—C11—N1	−179.0 (3)
C3—C4—C5—C6	0.6 (5)	C9—C10—C11—O1	179.4 (3)
C2—C1—C6—C5	0.4 (5)	C22—C10—C11—O1	0.5 (4)
C2—C1—C6—C7	−178.0 (3)	C8—C9—C12—C17	140.0 (3)
C4—C5—C6—C1	−1.1 (5)	C10—C9—C12—C17	−42.6 (5)
C4—C5—C6—C7	177.2 (3)	C8—C9—C12—C13	−43.3 (4)
C11—N1—C7—C8	−1.7 (4)	C10—C9—C12—C13	134.0 (3)
C11—N1—C7—C6	177.2 (3)	C17—C12—C13—C14	2.0 (5)
C1—C6—C7—N1	5.4 (4)	C9—C12—C13—C14	−174.8 (3)
C5—C6—C7—N1	−173.0 (3)	C12—C13—C14—C15	−1.3 (5)
C1—C6—C7—C8	−175.7 (3)	C20—O2—C15—C14	−169.1 (3)
C5—C6—C7—C8	5.9 (5)	C20—O2—C15—C16	10.8 (5)
N1—C7—C8—C9	−0.3 (4)	C13—C14—C15—O2	179.8 (3)
C6—C7—C8—C9	−179.1 (3)	C13—C14—C15—C16	−0.2 (5)
C7—C8—C9—C10	2.0 (4)	O2—C15—C16—C17	−179.1 (3)
C7—C8—C9—C12	179.5 (3)	C14—C15—C16—C17	0.9 (5)
C8—C9—C10—C11	−1.9 (4)	C13—C12—C17—C16	−1.3 (5)
C12—C9—C10—C11	−179.3 (3)	C9—C12—C17—C16	175.4 (3)
C8—C9—C10—C22	177.0 (3)	C15—C16—C17—C12	−0.1 (5)
C12—C9—C10—C22	−0.4 (5)	C11—O1—C18—C19	83.0 (3)
C7—N1—C11—O1	−177.6 (3)	C15—O2—C20—C21	174.2 (3)

### Hydrogen-bond geometry (Å, °)

**Table:**

*D*—H···*A*	*D*—H	H···*A*	*D*···*A*	*D*—H···*A*
C1—H1A···N1	0.93	2.41	2.758 (4)	102
C5—H5A···N2^i^	0.93	2.58	3.446 (4)	156
C13—H13A···N2^ii^	0.93	2.53	3.206 (4)	130

Symmetry codes:  (i) −*x*+1, *y*−1/2, −*z*+1/2; (ii) −*x*, *y*−1/2, −*z*+1/2.

## References

[bb1] Allen, F. H., Kennard, O., Watson, D. G., Brammer, L., Orpen, A. G. & Taylor, R. (1987). *J. Chem. Soc. Perkin Trans. 2*, pp. S1–S19.

[bb2] Bernstein, J., Davis, R. E., Shimoni, L. & Chang, N.-L. (1995). *Angew. Chem. Int. Ed. Engl* **34**, 1555–1573.

[bb3] Borgna, P., Pregnolato, M., Gamba, I. A. & Mellerio, G. (1993). *J. Heterocycl. Chem* **30**, 1079–1084.

[bb4] Bruker (2005). *APEX2*, *SAINT* and *SADABS* Bruker AXS Inc., Madison, Wisconsin, USA.

[bb5] Chantrapromma, S., Fun, H.-K., Suwunwong, T., Padaki, M. & Isloor, A. M. (2009). *Acta Cryst.* E**65**, o2914–o2915. 10.1107/S1600536809043943 PMC2971038 21578493

[bb6] Cosier, J. & Glazer, A. M. (1986). *J. Appl. Cryst.* **19**, 105–107.

[bb7] Flack, H. D. (1983). *Acta Cryst.* A**39**, 876–881.

[bb8] Fun, H.-K., Patil, P. S., Dharmaprakash, S. M. & Chantrapromma, S. (2008). *Acta Cryst.* E**64**, o1540–o1541. 10.1107/S1600536808021776 PMC2962165 21203245

[bb9] Goda, F. E., Abdel-Aziz, A. A.-M. & Attef, O. A. (2004). *Bioorg. Med. Chem* **12**, 1845–1852. 10.1016/j.bmc.2004.01.040 15051053

[bb10] Kamal, A., Khan, M. N. A., Reddy, K. S. & Rohini, K. (2007). *Bioorg. Med. Chem* **15**, 1004–1013. 10.1016/j.bmc.2006.10.027 17097292

[bb11] Malinka, W., Ryng, S., Sieklucka-Dziuba, M., Rajtar, G., Głowniak, A. & Kleinrok, Z. (1998). *Farmaco* **53**, 504–512. 10.1016/s0014-827x(98)00056-1 9836463

[bb12] Sheldrick, G. M. (2008). *Acta Cryst.* A**64**, 112–122. 10.1107/S0108767307043930 18156677

[bb13] Spek, A. L. (2009). *Acta Cryst* D**65**, 148–155. 10.1107/S090744490804362X PMC2631630 19171970

